# A novel stearic acid-modified hirudin peptidomimetic with improved pharmacokinetic properties and anticoagulant activity

**DOI:** 10.1038/srep14349

**Published:** 2015-09-24

**Authors:** Zhuguo Liu, Zheng Yu, Yuanyuan Huang, Yan Zhang, Guozhu Han, Xian Li, Mingxin Dong, Shuo Yu, Yu Wang, Jie Hu, Huiqin Guo, Yuanguo Cheng, Li Lv, Qiuyun Dai

**Affiliations:** 1 Beijing Institute of Biotechnology, Beijing 100071, China; 2 Dalian Medical University, Dalian 116044, Liaoning Province, China; 3 Beijing Institute of Microbiology and Epidemiology, Beijing 100071, China

## Abstract

A novel hirudin isoform 3 mimetic peptide, named peptide S2, has been prepared by introduction of a stearic acid modification. Peptide S2 exhibited superior inhibitory activity to hirulog-1 (Bivariludin) and showed significantly higher anticoagulant potency *in vivo*. Peptide S2 elevated the thrombin time, prothrombin time and activated partial thromboplastin time of rat and human plasma more efficiently than hirulog-1 and the unmodified form of peptide S2 (peptide **1**). Furthermore, peptide S2 inhibited arterial thrombosis and inferior vena cava in rat model 8 h after administration, and was 10-fold more potent than hirulog-1 300 min after administration of 0.1 μmol/kg peptide. The enhanced antithrombotic activity could be attributed to its long half-life (T_1/2_ = 212.2 ± 58.4 min), which was 13.1 and 14.7-fold longer than those of hirulog-1 (T_1/2_ = 15.1 ± 1.3 min) and peptide **1** (T_1/2_ = 13.5 ± 2.6 min), respectively. Further enzymatic degradation and binding assay with human serum albumin (HSA) demonstrated that the longer duration time should be originated from the slowing of trypsin or thrombin–mediated degradation, as well as its binding to HSA. The improved pharmacokinetic properties observed for peptide S2 has made it a promising therapeutic agent for the treatment of thrombi-related diseases.

Hirudins are naturally-occurring peptides found in the saliva of medicinal leeches, which can effectively inhibit the activity of thrombin in the blood coagulation pathway^1^. It appears that hirudins exist in several isoforms, including the recombinant hirudin isoform 1 (Refludan) that has been approved for treating patients with heparin-induced thrombocytopenia and related thromboembolic diseases^2^,^3^. By using the complementary interaction with the catalytic and anion-binding domains of thrombin, several hirudin-like peptidomimetic thrombin inhibitors have been developed^3^,^4^,^5^. For example, peptidomimetic hirulog-1 (*D*-FPRP-GGGG-QGDFEEIPEEYL, Bivalirudin or Angiomax^®^) contains a catalytic active binding domain (*D*-FPRP), an anion-binding exosite binding domain (QGDFEEIPEEYL) of hirudin isoform 1, and a linker of four Gly residues. Compared with heparin-combined glycoprotein IIb/IIIa inhibitors, hirulog-1 exhibits superior antithrombotic activity but with lower bleeding tendency^5^,^6^. This compound was first approved by FDA for percutaneous coronary intervention (PCI) in 2000 (2004 in Europe and Australia), and efforts to explore its anticoagulation effect in the cardiac and endovascular surgical settings also showed encouraging results^7^,^8^,^9^. However, the half-life of hirulog-1 in human plasma is extremely short (T_1/2_ = 20 ~ 25 min), which has dramatically limited the scope of its applications^5^. Consequently, hirulog-1 cannot be employed to treat diseases such as pulmonary and venous embolism.

It is known that modifying peptides with fatty acids can substantially improve their pharmacokinetic properties^10^,^11^, as exemplified by Liraglutide, a peptide drug for the treatment of type 2 diabetes. In this case, a fatty acid is attached to the ε-amino group of Lys^12^ of human GLP-1 peptide through a Glu spacer, ultimately affording a compound with half-life of 11–5 h^13^. Compared with PEG and human serum albumin modifications^14^, the fatty acid modification tends to produce medium prolongation of half-life.

We previously found a novel peptidomimetic thrombin inhibitor, named peptide **1** (Table 1), which is derived from the hirudin isoform 3, and it is more potent than hirulog-1 *in vitro*^15^. In an effort to obtain a peptide anticoagulant with high potency and desired half-life, we designed and synthesized the stearic acid-modified peptides based on peptide **1**. We coupled the ε-amino group of Lys residue with fatty acid, and used it to replace one of the Gly residues in the Gly linker consecutively, as shown in Table 1. The inhibitory experiments showed that peptide S2, in which the second Gly residue has been replaced with a modified Lys (stearic acid), exhibited anticoagulant activity comparable to hirulog-1. In the *in vivo* anticoagulant experiments, however, peptide S2 seems to be significantly more potent than hirulog-1 and peptide **1**. As a matter of fact, peptide S2 was found to substantially elevate the thrombin time (TT), prothrombin time (PT) and activated partial thromboplastin time (APTT) of rat and human plasma, affording a much longer duration time. As demonstrated by the rat arterial thrombosis model and inferior vena cava thrombosis model, peptide S2 is 10-fold more potent than hirulog-1, and can be maintained for more than 8 h. Theoretically, the enhanced anticoagulant activity of peptide S2 could be originated from its long half-life (T_1/2_ = 212.2 ±58.4 min) in rat blood, which is superior to hirulog-1 and peptide **1** under the same conditions (T_1/2_ < 16 min). To understand the intrinsic mechanism associated with the longer half-life of peptide S2, we have conducted degradation experiments mediated by thrombin and trypsin, as well as *in vitro* human serum albumin binding experiments. Overall, the much improved anticoagulant activity and pharmacokinetic properties of peptide S2 show promises for the development of peptide S2 as an effective and economic agent for various anticoagulation therapies.

## Results

### Modification of hirudin peptidomimetics with stearic acid

Based on the fact that hirudin isoform 3 is more potent than hirudin isoform 1, we previously designed and synthesized a novel hirudin mimetic peptide **1** (Table 1), which contains an anion-binding exosite binding domain (QGDFEPIPEDAYDE), a catalytic active binding domain ((*D*)-FPRP) and a four Gly linker. To maintain the anticoagulant activity of peptides while preventing thrombin and trysin from accessing the cleavage site, stearic acid modification was carried out on the linker of peptide **1**, primarily by substituting one of the Gly residues successively. All peptides prepared were more than 98% pure, and their molecular weight was determined by Ultraflex III TOF/TOF mass spectrometry (Bruker).

### Inhibitory activity to rat and human thrombin

As shown in Table 1, peptide S2 exhibited *in vitro* inhibitory activity to rat and human thrombin superior or similar to hirulog-1, but slightly inferior to peptide **1**. This result suggested that its binding to thrombin can be affected by introducing stearic acid. Notably, among the three peptides modified by stearic acid, peptide S2 seems to be the most potent compound that can inhibit rat and human thrombin. Hence, it was selected to be further evaluated in the *in vivo* anticoagulation experiments.

### Effects of peptides on the TT, PT and APTT of rat blood

**Figure 1:**
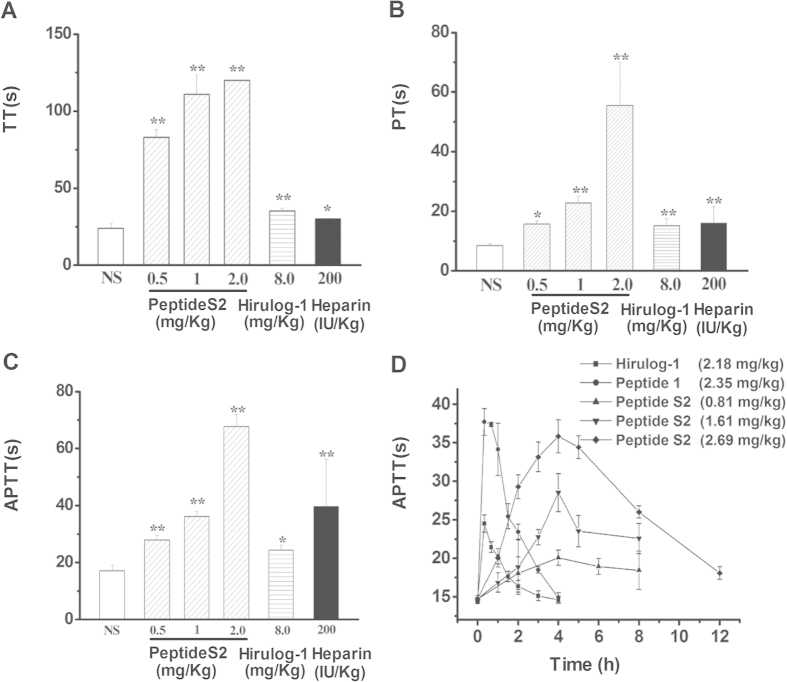
Effects of peptide S2 on TT, PT and APTT in rat plasma. n = 8, **p < 0.01, *p < 0.05, compared with saline group. (**A**) TT; (**B**) PT; (**C**) APTT; (**D**) The time course of APTT in rat plasma after peptide administration. (■)Hirulog-1, 2.18 mg/kg (1.0 μmol/kg); (•)Peptide **1**, 2.35 mg/kg (1.0 μmol/kg); (▲) Peptide S2, 2.69 mg/kg (1.0 μmol/kg); (▼) Peptide S2, 1.61 mg/kg (0.6 μmol/kg); (♦)Peptide S2, 0.81 mg/kg (0.3 μmol/kg).

As shown in Fig. 1, peptide S2 can significantly elevate TT, PT and APTT of rat blood 30 min after it was administered. Particularly, the antithrombotic activity of 0.5 mg/kg peptide S2 was found to be superior to the 8 mg/kg hirulog-1 and 200 IU/kg heparin. These dosages of hirulog-1 and heparin exhibited moderately high antithrombotic activity based on the results of preliminary experiments. As a matter of fact, the elevation of TT seems to be more distinctive than PT and APTT.

The APTT of rat plasma was determined at different time points after peptide injection, and the results are shown in Fig. 1D. Specifically, after the administration of peptide S2, the APTT increased in the first 4 h, and then decreased. And the antithrombotic activity was prolonged to be more than 8 h. In contrast, the APTT decreased sharply 1 h after hirulog-1 was administered^15^.

### Effects of peptides on PT, TT and APTT of human blood plasma

**Figure 2:**
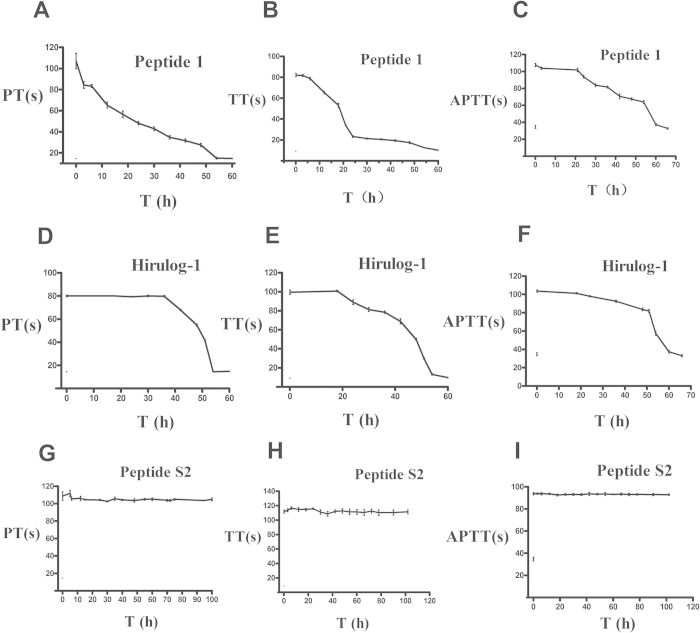
Effects of peptides on PT, TT and APTT of human blood plasma. Blood plasma samples were mixed with peptides (final concentration 2.1 μM) at 37 °C at different time points. TT, PT and APTT were determined accordingly. (**A**–**C**) Peptide **1**; (**D–F**) Hirulog-1; (**G**–**I**) Peptide S2.

When the peptide concentration was maintained at 2.1 μM, the coagulation parameters (PT, TT and APTT) of human blood plasma in the presence of peptide S2, peptide **1** and hirulog-1 were 102 h, 54 h and 54 h, respectively (Fig. 2). These results suggested that peptide S2 should possess improved pharmacokinetic properties comparing with hirulog-1 and peptide **1** in human blood plasma.

### Effects of peptides on rat carotid artery thrombosis

**Figure 3:**
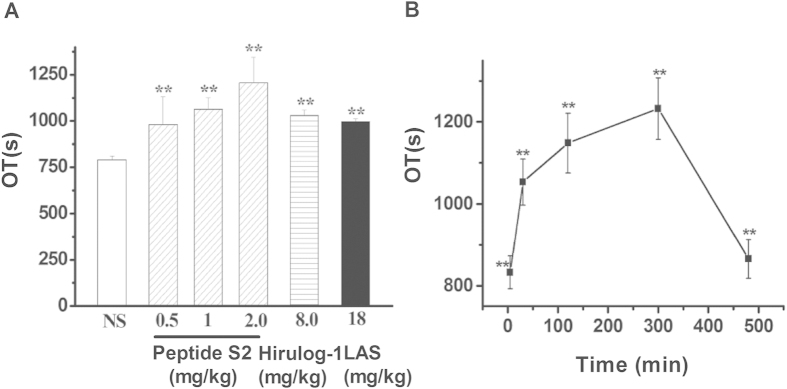
Effect of peptide S2 on the carotid artery thrombosis in rats. (**A**) OT values 30 min after the administration of 0.5, 1, 2 mg/kg peptide S2; (**B**) OT values at different time points after the injection of peptide S2 (1.0 mg/kg). Values were represented as means ± SD (n = 8). Significance of difference: **p < 0.01 compared with vehicle condition.

As shown in Fig. 3, peptide S2 (0.5, 1.0, 2.0 mg/kg) has induced a clear dosage-dependent increase in the occlusion time (OT). Specifically, 30 min after administration, the OT of rat carotid artery thrombosis was found to be 979.88 ± 150.61 s, 1063.75 ± 61.12 s, 1207.25 ± 137.82, respectively. Interestingly, the OT obtained after the administration of 1 mg/kg peptide S2 seems to be comparable to the one from 8 mg/kg hirulog-1 (1029.00 ± 29.81 s). On the other hand, the APTT of rat plasma after the administration of peptide S2 was found to be 8 h more than that after administration of hirulog-1 (Fig. 1D). Peptide S2 also showed prominent impact on the experimental arterial thrombosis (OT) during the first 8 h, indicating that the antithrombotic activity could be sustained for more than 8 h (Fig. 3B).

### Effects of peptides on the formation of inferior vena cava thrombosis (IVC)

As shown in Table 2, 20 min after the peptide was administered, the weight of wet thrombosis produced by hirulog-1 (1.0 μmol/kg) was 3.8 ± 2.8 mg, significantly lower than the one observed in the saline group (14.9 ± 7.9 mg). However, 300 min after the peptide administration, the anticoagulant activity of hirulog-1 seemed disappeared, and the weight of wet thrombosis was still as high as 15.3 ± 5.8 mg. Interestingly, 20 or 300 min after the administration of 0.1, 0.3, 0.6 and μmol/kg of peptide S2, the weight of wet thrombosis was found to be 1.7 ± 0.2 mg, 0, 0 and 0, respectively, suggesting that peptide S2 was more potent in inhibiting the formation of inferior vena cava thrombosis (IVC), and perhaps could persist for longer time. Overall, peptide S2 was about 10-fold more potent than hirulog-1 at 0.1 μmol/kg concentration.

### Effects on coagulation time (CT) and bleeding time (BT)

**Figure 4:**
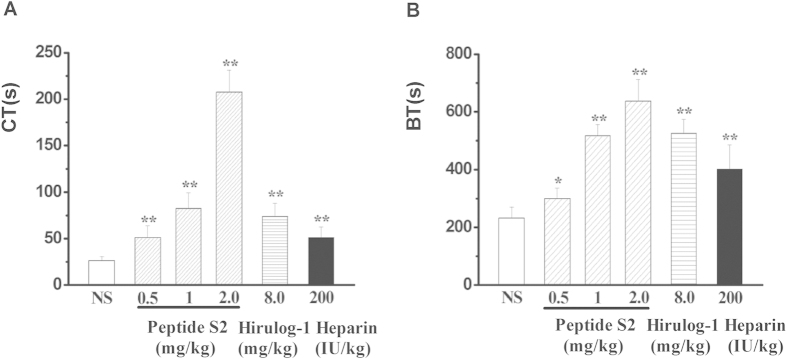
Effects of peptide S2 on rat coagulation time (**A**) and bleeding time (**B**). n = 8; ^**^p < 0.01, compared with saline group.

As shown in Fig. 4A, peptide S2 can significantly prolong the CT of rat blood. Consequently, the anticoagulant activity of 1.0 mg/kg of peptide S2 was found to be superior to the 8 mg/kg hirulog-1 and 200 IU/kg heparin.

To explore if any undesired side-effect(s) are present, the impact of peptide S2 on bleeding time (BT) has been closely examined. Specifically, after the administration of 0.5 mg/kg, 1.0 mg/kg and 2.0 mg/kg peptide S2 for 30 min, the bleeding time of rats was determined to be 300.00 ± 35.86 s, 517.50 ± 38.45 s, and 637.50 ± 74.79 s, as shown in Fig. 4B. Compared with the saline group, which has a bleeding time of 232.50 ± 38.45 s, this group showed 29.03%, 122.58% and 174.19% increase on the bleeding time. However, the low dose of peptide S2 (0.5 mg/kg) resulted in a shorter BT than the one obtained from the 8 mg/kg hirulog-1(BT = 525.00 ± 48.11 s) and 200 IU/kg heparin (BT = 401 ± 83.23 s).

### Pharmacokinetics of peptide S2 in rats

**Figure 5:**
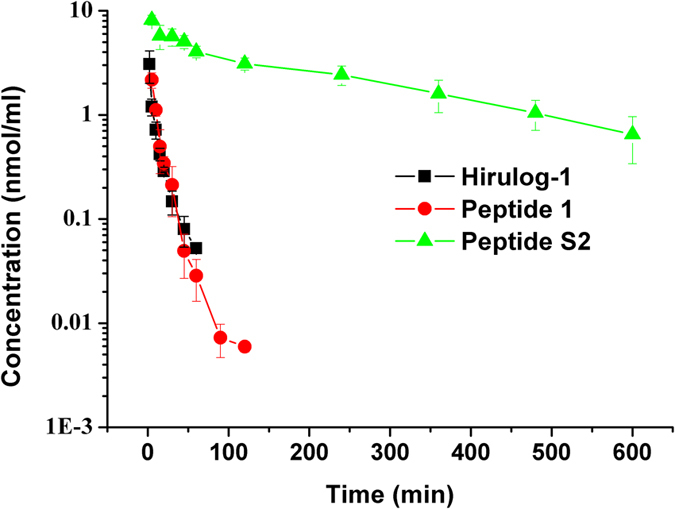
Peptide plasma concentration versus time after i.v. injection of 1 μmol/kg peptide (n = 5)(semi-log scale). (■) Hirulog-1; (●) Peptide **1**; (▲) Peptide S2.

The pharmacokinetic profiles of hirulog-1, peptide **1** and peptide S2 are presented in Fig. 5, in which the mean plasma concentration–time curves have been established. In addition, the mean pharmacokinetic parameters of hirulog-1, peptide **1** and peptide S2 obtained from the non-compartmental pharmacokinetic analysis are summarized in Table 3.

After the administration of 1 μmol/kg peptide S2 (i.v.), the AUC_(0-t)_ and AUC_(0-∞)_was found to be 1371.7 ± 207.8 and 1590.6 ± 311.7 nmol·min/ml, respectively, which were significantly higher than that obtained from hirulog-1 (23.7 ± 2.8 and 24.9 ± 2.7 nmol·min/ml) and peptide **1** (25.7 ± 2.6 and 25.9 ± 2.6 nmol·min/ml). Moreover, peptide S2 exhibited a terminal elimination half-life (T_1/2_ = 212.2 ± 58.4 min) significantly longer than hirulog-1 (15.1 ± 1.3 min) and peptide **1** (13.5 ± 2.6 min). These results clearly demonstrated that peptide S2 can maintain higher *in vivo* concentration than hirulog-1 and peptide **1**, which in turn would allow it to circulate longer in blood.

### Binding affinity of peptides to human serum albumin (HSA)

**Figure 6:**
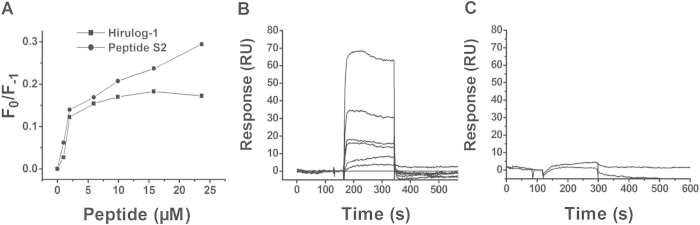
Interaction between HSA and peptide S2. (**A**) Effect of the intrinsic HSA fluorescence quenched by peptides. Excitation and emission wavelength was set at 282 and 336 nm, respectively. Fluorescence quenching was represented by the ratio of *F*_1_ to *F*_0_, in which *F*_1_ is the fluorescence intensity at a given peptide concentration, and *F*_0_ is the fluorescence in the absence of peptide. (**B**) Overlaid sensorgrams for peptide S2 bound to surface-immobilized HSA were obtained by surface Plasmon resonance analysis. The concentration of peptide S2 employed (from bottom to top) is 0, 0.1, 1.25, 2.5, 2.5, 5, and 10 μM, respectively. The running buffer is HBS-EP (0.01 M Hepes, pH = 7.4, 0.15 M NaCl, 3 mM EDTA, 0.005% surfactant). For each concentration, the peptide solution was first prepared in running buffer and then injected onto the surface of HSA and control antibody prepared over 3 min with a flow rate of 30 μL/min, followed by 15 min dissociation. (**C**) 0.1 μM and 10 μM (from bottom to top) hirulog-1 was allowed to bind to surface-immobilized HSA at a flow rate of 30 μL/min.

To understand why the half-life of peptide S2 has been largely prolonged, we next examined its ability to bind to human serum albumin (HSA) using intrinsic HSA fluorescence quenching analysis. The results are summarized in Fig. 6A. Apparently, the ratio of F_0_ to F obtained in the presence of peptide S2 was higher than that obtained in the presence of hirulog-1, suggesting that more peptide S2 was bound with HSA than hirulog-1. Furthermore, peptide S2 showed a dose-dependent fluorescence quenching within the range of 2.5 ~ 16 μM, whereas hirulog-1 exhibited a saturated binding with HSA in the low concentration area (~5 μM).

The kinetics involved in the association and disassociation of HSA to peptides was determined by a surface plasmon resonance method, in which the surface-immobilized HSA was employed, as illustrated in Fig. 6B. It showed that peptide S2 bound to HSA through a dose-dependent mode, and the bound peptide can quickly disassociate from the surface-immobilized HSA when the running buffer passed through the sensor. Figure 6C showed that hirulog-1 cannot effectively bind to HSA, neither did peptide **1** (data not shown), whereas peptide S2 can weakly bind to HSA.

### Resistance to degradation mediated by thrombin and trypsin

**Figure 7:**
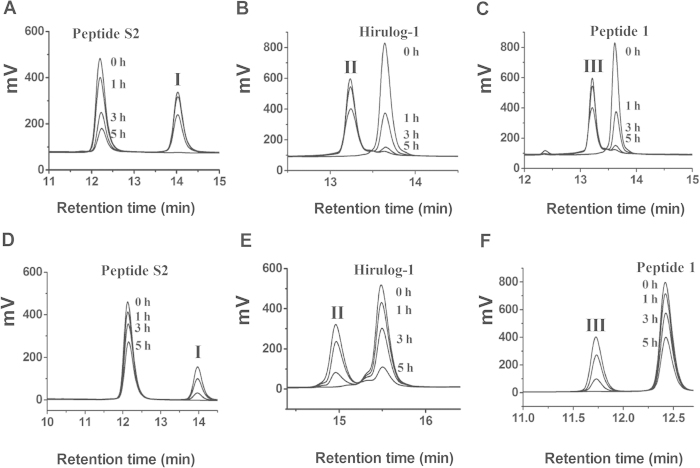
HPLC analysis of the degradation products mediated by trypsin (A–C) and human thrombin (D–E). (**A**) Cleavage of Peptide S2 by trypsin; (**B**) Cleavage of Hirulog-1 by trypsin (**C**) Cleavage of Peptide **1** by trypsin; (**D**) Cleavage of Peptide S2 by human thrombin; (**E**) Cleavage of Hirulog-1 by human thrombin; (**F**) Cleavage of Peptide **1** by human thrombin. The sequence of fragment I, II and III is PGK(stearic acid)GGQGDFEPIPEDAYDE-NH_2_, PGGGGQGDFEEIPEEYL and P-GGGG-QGDFEPIPEDAYDE-NH_2_, respectively. The elution gradients for HPLC analyses are described in Materials and Methods.

In the presence of 119 μg/ml trypsin, 39% and 23% of peptide S2 (0.79 mM) was intact after 3 h and 5 h incubation, as shown in Fig. 7A. However, at the same time points, only 10% and 6% of hirulog-1 remained (Fig. 7B). It also showed that peptide **1** was comparatively easier to be hydrolyzed, and only 5% and 1% of this peptide remained after 3 h and 5 h incubation (Fig. 7C). These results suggested that introducing a stearic acid group can partially protect the cleavage site of peptide S2 from trypsin degradation.

It is known that the catalytic binding domain (D)-FPRP can be slowly cleaved by thrombins^16^. Indeed, when human thrombin was employed (69 ΝΙΗ/ml), 93% and 69% of peptide S2 (0.41 mM) remained after 3 h and 5 h reaction, respectively (Fig. 7D). In contrast, only 57% and 23% of hirulog-1 were intact under the same conditions. Moreover, the degradation of peptide **1** was slower than hirulog-1, in which 70% and 46% of the starting peptide remained after 3 h and 5 h incubation, as shown in Fig. 7E. These results suggested that introducing stearic acid can partially protect the cleavage site of peptide S2 from the thrombin-mediated cleavage.

## Discussion

As illustrated in Figs 1 and 2, peptide S2 can significantly elevate the TT, PT and APTT of rat or human plasma, which indicated that it can last longer than hirulog-1 and peptide **1**. As a matter of fact, peptideS2 even inhibited the experimental arterial thrombosis and inferior vena cava 8 h after administration. Table 2 shows that peptide S2 was 10-fold more potent than hirulog-1 300 min after 0.1 μmol/kg peptide was administered. To the best of our knowledge, this is the first example of anticoagulation peptide obtained by fatty acid modification. So far, a number of sustained release systems for hirudin have been reported in the literature, such as the PEG-hirudin^17^, dextran coupled hirudin^18^, hydrogel based hirudin^19^ and cationic liposome delivery system^20^. However, they all suffer from undesired long anticoagulation time that often leads to the increased risk of bleeding, which makes them unsuitable to treat patients^21^. In contrast, peptide S2 exhibited an improved half-life (T_1/2_ = 212.2 ± 58.4 min) that is comparable to small molecule drugs such as argatraban^22^ and dabigatran (Pradaxa)^23^. The half-life of peptide S2 is similar to that of hirudin, but it is more easily produced and shows weak binding to thrombin. The improved pharmacokinetic properties of peptide S2 make it a promising intravenous injection agent, especially for the treatment of patients with pulmonary and venous embolism as a cost-effective agent with significantly reduced dosage.

It is known that trypsin and thrombin can cleave the Arg-Pro amino acid sequence in the catalytic active binding domain (*D*-FPRP)^16^. In this work, the prolonged half-life of peptide S2 could be mainly attributed to the introduction of stearic acid, which presumably hampers the cleavage of Arg-Pro sequence of *D*-FPRP domain. Indeed, the stearic acid-containing cleavage product was found at 14 min in the HPLC analysis (Fig. 7A,D). As shown in Table 1, the anticoagulant activity of peptides shows the following trend, peptide S1 < peptide S2 < peptide S3, which clearly demonstrated that the introduction of stearic acid mainly affected the interaction between *D*-FPRP and the catalytic active binding site of thrombin, rather than the anion binding exosite binding domain (QGDFEPIPEDAYDE*). Although the binding between peptide S2 and HSA did not seem to be very strong, and peptide S2 tended to disassociate from HSA quickly, such interaction still can alter the concentration of peptide S2 in blood circulation by reducing the efflux velocity from the renal glomerulus. The increased blood concentration of peptide S2 resulted in the higher anticoagulant activity of peptide S2 *in vivo*, even though peptide **1** showed higher inhibitory activity to thrombin *in vitro.*

Importantly, we found that a low dosage of peptide S2 showed a shorter bleeding time than that of hirulog-1. Specifically, when 0.5 mg/kg of peptide S2 was dosed, the bleeding time of rats was found to be 300.00 ± 35.86 s, which is significantly shorter than that induced by 8 mg/kg hirulog-1(525.00 ± 48.11 s) and heparin (100 IU/kg) (Fig. 4A). This result is consistent with the shorter coagulation time observed in the rat blood after the administration of 0.5 mg/kg peptide S2 (Fig. 4B). However, a high dose of peptide S2 (2 mg/kg) also resulted in a longer bleeding time (Fig. 4A). Therefore, a low dosage of peptide S2 is recommended since its high *in vivo* anticoagulant activity can be achieved under low dosage, as illustrated in Table 2.

In summary, we have designed and synthesized a novel hirudin isoform 3 peptidomimetic thrombin inhibitor (peptide S2) via stearic acid modification. This compound exhibited excellent *in vitro* and *in vivo* anticoagulant activity, and showed a significantly increased half-life (T_1/2_ = 212.2 ± 58.4 min). The prolonged half-life of peptide S2 could be mainly attributed to the introduction of stearic acid, which presumably prohibited the cleavage of Arg-Pro amino acid sequence in the presence of trypsin or thrombin. Overall, peptide S2 is an efficacious and economic agent for single intravenous injection, which should find applications in anticoagulation and therapeutic intervention of cardiovascular diseases.

## Methods

### Materials

Human thrombin and fibrinogen were purchased from National Institute for the Control of Pharmaceutical and Biological Products (Beijing, China). Chromozym TH (Tos-Gly-Arg-Pro-PNA) and Trypsin (modified sequencing grade) were obtained from Roche Corp (Indianapolis, USA). Kits for the determination of thrombin time (TT), prothrombin time (PT) and activated partial thromboplastin time (APTT) were purchased from Sunbio Lt. (Shanghai, China) and TECO (Neufahrn, Germany). HSA (human serum albumin, fraction V, fatty acid-free) and BSA (bovine serum albumin, fraction V) were obtained from Calbiochem (Merck, Germany). Rat thrombin was purchased from Sigma. Ethyl urethane, dl-lysine acetylsalicylate (LAS), heparin and pentoxifylline were purchased from Qingshengda Co. Ltd (Beijing, China), Harbin Pharmaceutical Group Co. Ltd. (Harbin, China), Aoboxing Biotechnology Co. Ltd (Beijing, China) and Shenyang First Pharmaceutical Factory (Shenyang, China), respectively. Other reagents (analytical purity) were purchased from common commercial vendors.

Sprague-Dawley (SD) rats (280–320 g or 180 ~ 220 g) from Beijing Animal Center or Animal Center of Dalian, Dalian Medical University, China were used in the APTT, TT, PT, CT, BT tests and venous thrombosis models. All animal experiments were carried out in accordance with approved guidelines and were approved by the Animal Care and the Use Committees of Beijing Institute of Biotechnology and Dalian Medical University.

### Peptide synthesis

All peptides were synthesized using solid-phase method on an Applied Biosystem 433 A Peptide Synthesizer. The stearic acid was introduced by Fmoc-Lys (stearic acid) in the synthesis of peptide-resin. After cleaving protecting groups, final peptide products were purified and characterized by analytical reversed-phase HPLC using the conditions described previously^15^,^24^. The primary sequences of peptides prepared are shown in Table 1.

### Inhibition of rat and human thrombin by peptides

The inhibition constant (*K*_*i*_) was determined as reported previously^15^. A 0.05 mL of peptide solution (concentration 10, 25, 50, 100, 150, 500 and 1000 nM, respectively) was added to 0.4 mL mixture of Hepes buffer (10 mM Hepes, 10 mM Tris, 0.1 M NaCl, 0.1% PEG 6000, pH 7.4) and bovine or human thrombin (0.25 NIH). The resulting solution was incubated at 25 °C for 2 min, followed by the addition of 0.05 mL Chromozym TH (concentration 25, 33, 40, 50, 100, 125, 200, 330, 500, and 1000 nM, respectively). The absorbance at 405 nm was measured using a Beckman DU640 spectrometer. The inhibition constant (*K*_*i*_) was determined using the following equation (Eq.1) originally developed for competitive inhibition processes^25^:


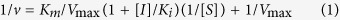


Herein, *S* is the initial substrate concentration; *I* is the inhibitor concentration; *v* is the initial steady-state velocity; *V*_max_ is the limiting maximal velocity; and *K*_*m*_ is the dissociation constant of the complex.

### Thrombin time (TT), prothrombin time (PT), activated partial thromboplastin time (APTT) and coagulation time (CT)

Forty-eight male SD rats (180 ~ 220 g) were randomly divided into six groups (n = 8) and anesthetized by *i.p.*20% ethyl urethane (1 g·kg^−1^). Subsequently, hirulog-1 (8.0 mg·kg^−1^), peptide S2 (0.5, 1.0, and 2.0 mg·kg^−1^), heparin (200 IU·kg^−1^) and saline were administrated to the rats via sublingual vein. Blood samples were obtained from the abdominal aorta, and then mixed with 3.8% sodium citrate solution in a ratio of 9:1 (v/v). The resulting samples were centrifuged at 3000 rpm for 15 min at 18 °C. TT, PT and APTT were determined according to the kit protocol (Sunbio Ltd., Shanghai) on a C2000 coagulometer (Precil, Beijing).

CT was obtained using the modified Dale’s method^26^. The dosage of peptide S2, hirulog-1, heparin and saline as well as the administration mode were identical to the ones employed in the bleeding time determination experiments. Thirty min after administration, blood samples were taken from the rat vein under eye ball with glass capillaries (20 μL), which were broken after 10 s, followed by a coagulation check. The time between blood extraction and blood coagulation in each capillary was recorded as coagulation time (CT).

### Determination of thrombin time (TT), prothrombin time (PT), activated partial thromboplastin time (APTT) of human plasma in the presence of peptides

The venous blood samples collected from healthy volunteers were mixed with 3.8% sodium citrate solution in a ratio of 9:1 (v/v), and then centrifuged at 3000 rpm for 15 min at 18 °C. At each point, 5 mL of the blood plasma on upper layer was allowed to mix with peptide solution (final concentration 2.1 μM) at 37 °C. Finally, TT, PT and APTT were determined according to the kit protocol (Sunbio Ltd., Shanghai) on a C2000 coagulometer (Precil, Beijing).

### Determination of APTT in rat plasma after peptide administration

Peptides were administrated to male SD rats (280–320 g) via tail veins. At different time points (0, 20, 40, 60, 90, 120, 180, and 240 min; or 0, 1, 2, 3, 4, 5, 8, and 12 h) after peptide injection, blood samples were collected via retroorbital vein plexus, and then mixed with 3.2% sodium citrate solution in a ratio of 9:1 (v/v). The resulting mixtures were centrifuged at 2,000 g for 15 min at 4 °C. The APTT of rat plasma was determined on a coagulation analyzer (Coatron M1, TECO, Germany) following the manual protocol. Briefly, a cuvette with 0.05 mL rat plasma was first warmed to 37 °C for 2 min, and then 0.05 mL of the warmed APTT reagent was added, followed by incubating at 37 °C for 3 min. Afterwards, 50 μL calcium chloride solution (0.25 M) was added into the cuvette, and the clotting time of plasma samples was recorded immediately with the coagulation analyser (APTT).

### Carotid artery thrombosis experiments

The carotid artery thrombosis in rats was induced by the method developed by Hladovec with minor modifications^12^. At first, male SD Rats (180 ~ 220 g) were randomly divided into 5 groups (n = 8) and anesthetized by *i.p.* 20% ethyl urethane (1 g/kg). The right carotid artery was first carefully separated to give a 1.5 cm long open access, and then the stimulating electrode and the temperature electrode of BT 87–3 thrombosis radiometer (Baotou medical college, China) were linked to both ends of the artery. Peptide S2 (0.5, 1.0, and 2.0 mg/kg), hirulog-1 (8.0 mg/kg), LAS(18 mg/kg) and saline were administered via sublingual vein, followed by a continuous electric stimulation (1.6 mA, 7 min) that started 30 min after injection. The time from the initiation of electric stimulation to the sudden drop of carotid temperature was recorded as occlusion time (OT), or thrombus formation time. In the time-effect relationship study of *i.v.* peptide S2, the OT has been measured at 30 min, 120 min, 300 min and 480 min after injection (1.0 mg/kg).

### Inferior vena cava thrombosis experiments

Thrombosis of rats (280 ~ 320 g, half males and half females) was induced by inferior vena cava (IVC) ligation, which can produce a stasis thrombus as previously reported^27^,^28^. Specifically, a laparotomy with ligation of the IVC below the renal veins and all visible side branches was first performed. 20 min or 5 hours after the administration of peptide or saline, the thrombosis in the IVC was carefully dissected and weighted after removing blood residual.

### Bleeding time test (BT)

Bleeding time was determined as reported previously^29^. Male SD rats (180–220 g) were divided into 6 groups (n = 8). Saline, peptide S2 (0.5, 1.0, and 2.0 mg/kg), hirulog-1 (8.0 mg/kg) and heparin (200 IU/kg) was administered via sublingual vein, respectively. Thirty minutes after the injection, bleeding was induced by tail transection of the anesthetized rat with a size 21 disposable scalpel blade 2 mm from the tail tip. The bleeding tail was wiped with filter paper every 30 s, and the bleeding time was recorded when no blood was observed on the filter paper.

### Pharmacokinetics studies

SD Rats were randomly divided into three groups (n = 5). Subsequently, 1 μmol/kg hirulog-1, peptide **1** and peptide S2 were injected intravenously, and blood samples were collected through the jugular vein (0.3 mL)^30^,^31^ in 1.5 mL heparinized micro-centrifuge tubes at the following time point: hirulog-1: 0, 2, 5, 10, 15, 20, 30, 45, 60, and 90 min; peptide1: 0, 5, 10, 15, 20, 30, 45, 60, 90 and 120 min; peptide S2: 0, 5, 15, 30, 45, 60, 120, 240, 360, 480 and 600 min. All blood samples were immediately centrifuged at 5000 rpm for 5 min at room temperature, and the plasma obtained was stored at −20 °C before analysis. To the rat plasma (100 μL) was added 10 μL internal standard (80 ~ 100 ng/ml Carbamazepine), followed by mixing with 200 μL methanol with vortexing for 1 min. At this stage, certain amounts of proteins may precipitate out, thus this mixture was centrifuged at 12,000 rpm for 5 min at room temperature to achieve complete separation. The 20 μl supernatant was injected into a HPLC (Symbiosis, Spark, Holland; column: 50 mm × 2.1 mm, 5 μm, Agilent Eclipse Plus) –MS/MS system (ABI 4000 Q-Trap triple quadrupole mass spectrometer) equipped with electrospray ionization (ESI) source in the positive ion mode (Foster City, USA). The HPLC elution gradient was as follows: 0–0.5 min, 5–5% (B); 0.5–3 min, 5–95% (B); 3–4 min, 95–95% (B); 4–4.06 min, 95–5% (B); 4.06–6 min, 5–5% (B); the flow-rate was 0.4 ml/min; A was 0.1% formic acid/Waterl, B was 0.1% formic acid/methanol. The MS parameters were: CUR10Psi; Ion Spray Voltage (IS) 5500v; Collision activated dissociation (CAD) High, Gas1 = 50 psi, Gas2 = 50 psi, TEM = 500 °C. The transitions of hirulog-1, peptide 1 and peptide S2 for the multiple reaction monitoring (MRM) were 727.7 → 943.8, 784.1 → 1102.4 and 896.9 → 926.4, respectively.

The LOQ (Limit of Quantitation) of hirulog-1, peptide **1** and peptide S2 were 100, 10, 200 ng/ml with S/N above 10, respectively. The LOD (Limit of Detection) of hirulog-1, peptide **1** and peptide S2 were 30, 3, and 40 ng/ml at a S/N of 3:1, respectively. The QC samples of hirulog-1, peptide **1** and peptide S2 were 200, 2500, 16000 ng/ml, 25, 500, 6500 ng/ml and 500, 2500, 40000 ng/ml, respectively. The intra-assay accuracy of hirulog-1, peptide **1** and peptide S2 varied between 99.7 and 105.2%, 90.67 and 97.25%, 96.90 and 106.82%, respectively, and the precision deviations were less than 11.87%, 4.43%, 9.44%, respectively. The inter-assay accuracy of hirulog-1, peptide **1** and peptide S2 ranged from 98.33 to 102.47%, 90.67 and 97.25%, 104.91 and 106.76%, respectively; the precision deviations were less than 11.78%, 6.79%, 10.16%, respectively. The mean absolute recovery rates of hirulog-1, peptide **1** and peptide S2 were 80.13%, 58.01%, 71.25%, respectively. All three peptides remained stable in plasma during three freeze/thaw cycles, at room temperature for at least 2 h. The supernatant of plasma samples were also found to be stable for at least 24 h at 10 °C in the autosampler.

The pharmacokinetic properties of hirulog-1, peptide **1** and peptide S2 can be evaluated by analyzing the corresponding plasma concentration–time profiles, which have been obtained by calculating with Microsoft Excel 2003 and on-compartment analysis^32^. The statistical significance of the difference among pharmacokinetic parameters was determined using the mean comparison Student’s t-test, and the resulting P values were lower than 0.05, which is considered to be statistically significant.

### Binding of peptide S2 to human serum albumin

#### Fluorescence quenching method

The binding of peptide S2 to human serum albumin (HSA) was determined using an intrinsic fluorescence quenching method^33^. Fluorescence measurements were conducted on a Hitachi F-4500 spectrofluorometer with a slit width of 5 nm (excitation) and 5 nm (emission). All measurements were performed at 25 °C in a 1 cm path length quartz cell. Samples were excited at 280 nm and the fluorescence intensity was measured between 300–400 nm. Human serum albumin (5 μM) was dissolved in 10 mM HEPES/100 mM NaCl solution (pH 7.4), and then titrated with the stock solution of peptides prepared in the same buffer. The background fluorescence spectra were recorded and subtracted from the ones collected from the peptide-containing samples.

### Surface Plasmon Resonance (SPR) analysis

The binding of peptide S2 and hirulog-1 to HSA was measured by SPR method using a Biacore 3000 system (GE Healthcare) as previously described^34^. Briefly, HSA was first immobilized on CM5 sensor chips using the standard amine coupling protocol provided by manufacturer. The carboxymethylated dextran surface was then activated by injecting 45 μL of the solution containing 0.2 M *N*-ethyl-*N*’-(3-dimethylaminopropyl) carbodiimide hydrochloride and 0.05 M *N*-hydroxy succinimide. Subsequently, HSA and the control antibody were dissolved in 10 mM Na-citrate solution (pH 5.0, 50 μg/ml) and then injected until the desired amount of HSA and control antibody were coupled (8000 RUs). At this point, a buffer with higher ionic strength (HBS-EP, 0.01 M Hepes, pH = 7.4, 0.15 M NaCl, 3 mM EDTA, 0.005% surfactant) was used to wash off the ligands bound non-covalently. Finally, the 1 M ethanolamine hydrochloride solution (pH 8.5) was employed to cap the free NHS esters present on the sensor surface. Different concentrations of peptide S2 (0.1 ~ 10 μM) and hirulog-1(0.1 μM and 10 μM) were prepared in the HBS-EP buffer. All samples were injected onto the surface of HSA and control antibody over 3 minutes at a flow rate of 30 μL/min, followed by a dissociation period of 15 min. The 2.5 μM sample was subject to this protocol twice. Sensorgrams were obtained for each peptide concentration, respectively.

### Degradation of peptides mediated by trypsin and thrombin

The degradation of peptides mediated by trypsin can be effectively monitored by HPLC analysis. Specifically, peptide S2 (0.79 mM) was allowed to incubate with trypsin (119 ng/μL) in the Tris-HCl buffer (90 mM, pH = 8.5) at 37 °Cfor 0 h, 1 h, 3 h and 5 h, respectively. At each time point, 6 μL reaction mixture was injected into the HPLC system (Kromasil C_18_ column, 4.6 × 250 mm) and eluted by a gradient of solvent A (0.1% TFA/water) and B (0.1% TFA/acetonitrile) (0 ~ 1 min, 5 ~ 55%B; 1 ~ 25 min, 55% ~ 80%B) with a flow rate of 1 ml/min. The detector wavelength was set at 214 nm. Similar reaction and analytical conditions were employed for the degradation studies of hirulog-1 and peptide **1**, except that a different HPLC elution gradient has been used (0 ~ 20 min, 5% ~ 50%B).

The catalytic cleavage of peptides mediated by thrombin was also examined using the same method. Specifically, a peptide solution (0.41 mM) was first incubated with thrombin (69 NIH/mL) in Tris-HCl buffer (70 mM Tris HCl, NaCl 50 mM, pH = 8.0) at 37 °C. After 0 h, 1 h, 3 h and 5 h, 12 μL of the reaction mixture was subject to HPLC analysis at a flow rate of 1 ml/min with different elution gradients (peptide S2: 0 ~ 1 min, 5 ~ 55% B; 1 ~ 25 min, 55% ~ 80% B; hirulog-1: 0 ~ 20 min, 5% ~ 80% B; peptide **1**: 0 ~ 25 min, 5% ~ 80% B).

### Statistical analysis

The statistical analysis was performed using SPSS 11.5 software. Results were expressed as the mean ± SD; data were analyzed using either the unpaired t-test, or the one-way analysis of variance (ANOVA), followed by the Student–Newman–Keul’s test. Generally, difference with a p-value < 0.05 is considered statistically significant. In this work, n represents the number of animals tested in a single experiment.

## Additional Information

**How to cite this article**: Liu, Z. *et al.* A novel stearic acid-modified hirudin peptidomimetic with improved pharmacokinetic properties and anticoagulant activity. *Sci. Rep.* **5**, 14349; doi: 10.1038/srep14349 (2015).

## Figures and Tables

**Table 1:** Inhibition constant for peptides (Ki, nM) in rat and human thrombin–catalyzed hydrolysis of Chromozym TH.

**Peptide**	**Molecular Weight(Da)**	**Sequence**	***K*_*i*_ (nM)**^a^ **Rat Human**	
Peptide 1	2348.03	*D*-FPRP-GGGG-QGDFEPIPEDAYDE^*^	13.40 ± 0.46	5.80 ± 0.55^b^
Peptide S1	2687.02	*D*-FPRP-K(stearic acid)GGG-QGDFEPIPEDAYDE^*^	37.42 ± 1.16	19.48 ± 0.40
Peptide S2	2687.02	*D*-FPRP-GK(stearic acid)GG-QGDFEPIPEDAYDE^*^	15.24 ± 0.18	11.75 ± 0.46
Peptide S3	2687.02	*D*-FPRP-GGGK(stearic acid)-QGDFEPIPEDAYDE^*^	54.49 ± 0.82	10.95 ± 0.15
Hirulog-1	2179.31	*D*-FPRP-GGGG-QGDFEEIPEEYL	20.50 ± 0.62	11.77 ± 0.09^b^

^*^Peptides are C-terminally amidated.

^a^All inhibition constants (*K*_*i*_) were determined by the following equation: 1/*v* = *K*_*m*_/*V*_max_ (1 + [*I*]/*K*_*i*_)(1/[*S*]) + 1/*V*_max_.

^b^Excerpted from a previously published study^15^.

**Table 2:** Inhibitions of the IVC thrombosis formation in rats by peptide S2.

**Peptide**	**Dosage (mg/kg)**	**Time (min) after administration**	**Weight of wet thrombosis (mg)**
Saline	0	20	14.9 ± 7.9
Hirulog-1	2.18	20	3.8 ± 2.8
Hirulog-1	2.18	300	15.3 ± 5.8
Peptide S2	2.69	20	1.3 ± 1.8
Peptide S2	2.69	300	0
Peptide S2	1.61	300	0
Peptide S2	0.81	300	0
Peptide S2	0.27	300	1.7 ± 0.2

**Table 3:** Pharmacokinetic parameters of anticoagulant peptides injected at 1 μmol/kg i.v. (n = 5).

**Parameter**	**Hirulog-1**	**Peptide 1**	**Peptide S2**
AUC_(0-t)_ (nmol·min/ml)	23.7 ± 2.8	25.7 ± 2.6	1371.7 ± 207.8^*^
AUC_(0-∞)_ (nmol·min/ml)	24.9 ± 2.7	25.9 ± 2.6	1590.6 ± 311.7^*^
MRT_0-t_ (min)	12.1 ± 2.1	13.9 ± 1.1	197.4 ± 14.3^*^
CL (ml/kg/min)	42.6 ± 5.2	36.4 ± 3.6	0.7 ± 0.1^*^
V_ss_ (ml/kg)	521.7 ± 150.9	503.9 ± 21.7	145.6 ± 15.5^*^
T_1/2_ (min)	15.1 ± 1.3	13.5 ± 2.6	212.2 ± 58.4^*^
C_max_ (nmol/ml)	3.1 ± 1.1	2.3 ± 0.4	10.1 ± 1.1^*^

^*^p < 0.05 compared with hirulog-1 and peptide **1**, respectively.
